# Differentiating Stages of Bipolar and Unipolar Depression—The Possible Role of sICAM-1 and sVCAM-1

**DOI:** 10.3390/cells13141213

**Published:** 2024-07-18

**Authors:** Maja Pantovic-Stefanovic, Natasa Petronijevic, Bojana Dunjic-Kostic, Milica Velimirovic, Vladimir Jurisic, Tatjana Nikolic, Sara Dodic, Maja Ivkovic

**Affiliations:** 1 Department of Bipolar Disorders, Clinic for Psychiatry, University Clinical Centre of Serbia, Pasterova 2, 11000 Belgrade, Serbia; majapantovic@yahoo.it (M.P.-S.);; 2 School of Medicine, University of Belgrade, Dr Subotica 8 st., 11000 Belgrade, Serbia; natasapetronijevic@yahoo.com (N.P.);; 3 Institute of Clinical and Medical Biochemistry, Pasterova 2, 11000 Belgrade, Serbia; 4 Faculty of Medical Sciences, University of Kragujevac, Svetozara Markovica 69, 11000 Kragujevac, Serbia

**Keywords:** mood disorders, bipolar disorder, depression, inflammation, adhesion molecule

## Abstract

Increased immune–inflammatory activation has been repeatedly linked to etiopathogenesis and the progression of both major depressive disorder (MDD) and bipolar depression (BD). We explore the role of soluble intercellular cell adhesion molecule-1 (sICAM-1) and soluble vascular cell adhesion molecule-1 (sVCAM-1) in diagnostic differentiation and disorder progression in patients with MDD and BD. Serum levels of sICAM-1 and sVCAM-1 were measured in 137 patients (MDD = 93 and BD = 44) and compared with 73 healthy controls. The severity of psychopathology was assessed using the Hamilton Depression Rating Scale and Clinical Global Impression Scale. After adjustment for multiple confounders, we noticed significant downregulation of sVCAM-1 and upregulation of sICAM-1 levels in both patient groups. Decreased sVCAM-1 levels were detected in patients with acute episodes of BD when compared to MDD. Immune mediators were related to indicators of progression in both mood disorders. They also followed different post-treatment normalization patterns in MDD and BD and in relation to the stage of each disorder. Adhesion molecules could potentially be useful in discriminating between patients with MDD and BD and determining the possible progression of the disorders. Future nosological methods should include time-dependent pathoplasticity and biological correlates, at least for affective disorders.

## 1. Introduction

Increased immune–inflammatory activation is one of the factors repeatedly linked to the etiopathogenesis of mood disorders, regardless of their polarity. Imbalances in numerous immune mediators have been linked not only to major depressive disorder (MDD) and bipolar disorder, but also to depression symptoms in patients with somatic comorbidity and even in healthy adult populations [1,2,3,4,5]. More importantly, owing to the shared depressive symptoms and frequently unreported or delayed onset of manic or hypomanic episodes in bipolar disorder, patients with bipolar depression (BD) are often mistakenly diagnosed with MDD. This could increase the risk of both drug-induced mania and increased frequency of affective episodes and, considering the immune-inflammatory activation in both disorders, cause further progression. The connection between inflammation and MDD, however, remains unclear and inconclusive in either direction [6]. Thus, a better understanding of immune mediators that could possibly help differentiate between unipolar- and bipolar-type depression would lead to timely and adequate treatment, as well as enhance neuroprotection and suppress neurodegeneration, particularly in bipolar disorder [1,6]. Previous studies indicate that soluble vascular cell adhesion molecule-1 (sVCAM-1) and soluble intercellular adhesion molecule-1 (sICAM-1) can serve as markers of clinical presentation of mood disorders and their neurobiological manifestations [7,8]. ICAM-1 and VCAM-1, as transmembrane proteins, are reportedly expressed on cerebral vascular endothelial cells, neurons, astrocytes, and microglial cells and are broadly distributed in the matured brain. These molecules play a pivotal role in navigating the movement of leukocytes, and their imbalance may influence blood–brain barrier (BBB) permeability [9,10,11,12]. By increasing traffic of immune cells to the CNS, they build a bridge between peripheral and neurological inflammation in severe mental illness. In response to inflammation, endothelial cells boost selectins to decelerate leukocytes before they firmly adhere using integrins, as well as ICAM1 and VCAM1. Leukocytes express integrin receptors that connect with CAMs, helping them move through the BBB, an activity observed to increase in schizophrenia [9,13,14]. Adhesion molecules maintain cellular structural integrity and assist in the process by which leukocytes move out of blood vessels. ICAM1 and VCAM1 can be shed from cells (T cells, macrophages, endothelial cells, or glial cells) [15] and released into circulation through various processes. These soluble forms allow other immune mediators to enter the CNS and modulate monoaminergic transmission [16,17,18,19].

Such neuromodulatory events can impair normal neurotransmission and induce changes in psychopathology [20,21], potentially leading to affective symptoms. Studies report contrasting results on imbalances in adhesion molecules, such as ICAM-1 and VCAM-1, in patients with both bipolar disorder and MDD, with the majority of them indicating a potential link between immune dysregulation and the development and progression of these conditions [20,21]. Patients with major depressive disorder typically exhibit higher levels of adhesion molecules related to proinflammatory conditions like ICAM-1, while those with bipolar disorder show an increase in anti-inflammatory molecules like VCAM-1 [7,17,22]. There is also evidence supporting the association between both sICAM-1 and sVCAM-1 and the clinical course and progression of MDD [17,22,23]. Also, while the effects of psychotropic drugs on ICAM-1 and VCAM-1 are not fully elucidated, existing evidence suggests these medications, particularly antidepressants, could modulate the expression of these adhesion molecules, potentially contributing to their therapeutic efficacy and side effect profiles in bipolar disorder and MDD [24,25,26]. This implies sICAM-1 and sVCAM-1 may be important in mood disorder pathophysiology, particularly depressive symptoms, or even help to discriminate between patients in both unipolar and bipolar depressive episodes. To our knowledge, their levels have not been a subject of investigation in these domains.

This investigation aimed to assess changes in the imbalance of sICAM-1 and sVCAM-1 in MDD and BD and in comparison to a healthy control group. Further, we sought to explore the relationship between possible immune alterations, psychopathology, and clinical characteristics, including disease progression and treatment application in patients with different polarities of depression.

## 2. Materials and Methods

### 2.1. Patients and Design

The sample size required to obtain the power of 1 − β = 0.80 at α = 0.05 was assessed based on similar previous studies [27]. For determining inter-group differences, twenty-two subjects per group were sufficient. A total of 189 patients hospitalized at the Clinic for Psychiatry, University Clinical Centre of Serbia, Belgrade for the treatment of acute episodes of depression (MDD = 131 and BD = 58) over a one-year period initially provided informed consent to participate in the study. The final sample of 137 patients (MDD = 93 and BD = 44) with bipolar or unipolar depression was derived after subsequent screening for inclusion/exclusion criteria. This prospective study was conducted in accordance with the Declaration of Helsinki and approved by the Ethics Committee of the University Clinical Centre of Serbia (protocol code 622/1 and 4072/5 and 24.1.2022 and 24.08.2012). Psychiatric diagnosis was confirmed based on DSM-5 criteria for MDD and depressive episodes of bipolar disorder [28]. The Mood Disorder Questionnaire was also used to confirm depression polarity [29]. Severity of depressive symptoms was assessed with the Hamilton Depression Rating Scale (HDRS), while the presence of manic symptoms was measured by the Young Mania Rating Scale (YMRS) [30,31]. Global illness severity was assessed by the Clinical Global Impression Scale—Severity (CGI-S) [32,33]. All psychometric scales used to assess the severity of symptoms and level of functioning were applied two times—upon admission and at remission, before hospital discharge.

The clinical staging model proposed by Kapczinski et al. [34] was used to classify the patients into early vs. late stages of the disorder: Stage 0—at risk, positive family history, mood, or anxiety symptoms; Stage I—well-defined periods of euthymia without symptoms; Stage II—interepisodic symptoms related to comorbidities; Stage III—marked impairment in cognition or functioning; and Stage IV—unable to live autonomously due to impairment. The staging criteria took into account interepisode clinical presentation and recovery, functioning, impairment comorbidities, and cognitive parameters. Patients in Kapczinski Stage I and Stage II were classified as patients in the early stage of mood disorder, whereas patients in Kapczinski Stage III and Stage IV were classified as being in the late stage of the disorder. Two trained psychiatrists evaluated the diagnoses and conducted psychometric evaluations using detailed semi-structured interviews. Charts and complete medical records of each enrolled patient were reviewed by a blinded research assistant in order to obtain all clinical data concerning the course of illness and treatment with mood stabilizers (MSs) or antidepressants (ADs).

Inclusion criteria for patient groups were: (a) admission HDRS ≥ 18 points; (b) admission CGI-S ≥ 4 [35,36]; (c) admission YMRS ≤ 8 [37]; (d) remission criteria HDRS ≤ 8 and YMRS ≤ 8 [31,33].

Exclusion criteria for the patient groups were: (a) being treated with mood stabilizers, antipsychotics, or antidepressants for at least four weeks prior to hospitalization [22]; (b) a history of any other psychiatric illness according to DSM-5 disorders; (c) a history of poor responses to previous treatment with MSs and ADs; (d) >8 mood episodes in the previous year; (e) medical disorders related to changes in the inflammatory response [38]; (f) socio-demographic properties previously linked to alterations of immune mediators [8,38]: obesity (body mass index (BMI) ≥ 30 kg/m^2^) or undernutrition with recent weight loss and heavy smoking (≥20 cigarettes/day); (g) the current use of therapy frequently related to immunomodulation (i.e., corticosteroids, acetylsalicylic acid, nonsteroidal anti-inflammatory medications, immunosuppressors); (h) the presence of an acute infection assessed through a complete physical examination, blood and urine analysis, and body temperature quantification.

Based on confounding factors, the healthy control subjects were directly invited through the method of convenient sampling (personal network) to participate in the study. Healthy controls were recruited from an initial sample of 89 consenting full-time working individuals with different vocations and residing in urban or rural areas. The controls were also screened for psychiatric disorders using the Structured Clinical Interview for DSM-5 (SCID 5).

Inclusion criteria for the healthy control group were: (a) absence of psychiatric disorders confirmed with the Structured Clinical Interview for DSM-5 (SCID 5).

Exclusion criteria for the healthy control group were: (a) criteria (e) and (f) from the patient group; (b) the use of any psychotropic or other medications four weeks prior to blood withdrawal; and (c) a first-degree relative with a history of major psychiatric disorders. Lastly, based on the selection criteria, seventy-three consenting healthy individuals were matched with patients with BD for the most relevant potential confounding factors [31,36]: age, BMI, and smoker/non-smoker status.

### 2.2. sICAM-1 and sVCAM-1 Measurements

Blood samples were collected a day after hospital admission for the acute episodes and a day before hospital discharge for patients that followed the remission according to study criteria. For serum preparation, whole blood samples were collected in the morning, between 7 a.m. and 8 a.m., after overnight fasting. The blood was allowed to clot by leaving it undisturbed at room temperature for 30 min, followed by an additional 30 min at 4 degrees Celsius. Afterwards, the tubes were centrifuged for 15 min at 3000 rpm, allowing serum separation. Until used, serum was stored at −80 degrees Celsius.

ICAM-1 and VCAM-1 were measured in serum samples using a sandwich ELISA test produced by Abcam. As described in the manufacturer’s protocol, the plates needed to be covered with capture antibodies, followed by the addition of samples (standards and human serum samples run in duplicates) and biotinilated detection antibodies, forming an antibody–antigen–antibody structure. Adding streptavitin-HRP and TMB produced a blue color, the saturation of which depended on ICAM-1/VCAM-1 levels. The absorbance was read at 450 nm using a microplate reader. A standard curve was used to calculate the levels of ICAM-1/VCAM-1. All biochemical measurements were conducted by investigators who were blind to the patients’ diagnostic states. The measurement procedures and methods have been described in detail previously [39,40].

### 2.3. Statistical Analysis

The Software Package for Social Sciences for Windows v.19.0 (SPSS Inc., Chicago, IL, USA) and G*Power 3.1 software were used to analyze the data. Normal distribution was assessed using the Kolmogorov–Smirnov test. We used standard descriptive statistics (i.e., frequencies and percentages for attributive variables and mean value with standard deviation for numeric variables) to describe the data. For discrete variables, the chi-square test (χ^2^) and χ^2^ with continuity correction according to Yates or Fisher’s exact probability test were used to analyze the data. Depending on the normality of the data and the number of groups, the independent *t*-test, Mann–Whitney U test, or one-way analysis of variance (ANOVA) was used for continuous variables. Partial correlation coefficients were used to evaluate associations between sICAM-1/sVCAM-1 levels and clinical characteristics of patients in the acute stage and in remission, adjusted for sex, age, BMI, and smoking status. Binary logistic regression was performed in order to identify sICAM-1/sVCAM-1 as potential predictors of the disorders, with sex, age, BMI, and smoking status as confounding factors. To further assess the differences in adhesion molecule levels between the study groups, we used multivariable analysis (ANCOVA) only after adjusting for potential confounding factors (age, gender, BMI, and smoker/non-smoker status). Based on the number of groups and the initial results of ANCOVA testing, multiple comparison analysis was carried out via Fisher’s least significant difference (LSD) test. To additionally increase data precision, we used the bootstrapping method throughout the analysis. Statistical significance was confirmed only if both *p*-values were ≤0.05 and the confidence intervals (CI) did not contain zero.

## 3. Results

The sociodemographic and clinical characteristics of the study and control groups are shown in Table 1.

### 3.1. Association between sICAM-1 and sVCAM-1 and Clinical Characteristics of Patients with MDD and BD

The partial correlation coefficients describing associations between clinical characteristics of patients and adhesion molecule levels are presented in Table 2. A negative correlation of a medium strength between sVCAM-1, the number of previous mood episodes (r = −0.272, *p* = 0.008, CI: −0.421–−0.091), and the number of previous inpatient treatments (r = −0.230, *p* = 0.027, CI: −0.391–−0.026) was found in the samples of patients in acute depressive episodes, regardless of the type of polarity. The levels of sICAM-1 showed no association with the clinical characteristics of the whole sample.

Further subsample analyses revealed that higher sICAM-1 levels in BD were associated with more intense acute depressive symptoms measured by HAMD (r = 0.357, *p* = 0.041, CI: 0.017–0.647). sICAM-1 was also higher in patients with a positive family history for BD (family history of BD: 640.42 ± 159.69; no family history of BD: 333.60 ± 26.97, *p* = 0.013, CI: 146.709–454.611) in remission. Similarly, sVCAM-1 in the acute episodes was lower in patients with a hereditary load for BD (family history of BD: 397.32 ± 273.24; no family history of BD: 772.95 ± 44.18, *p* = 0.010, CI: 205.324–533.116).

On the other hand, in the subsample of patients with acute MDD, ICAM-1 showed a strong positive association with a longer duration of the disorder (r = 0.394, *p* = 0.001, CI: 0.222–0.561) and the duration of the current episode (r = 0.317, *p* = 0.009, CI: 0.103–0.513).

Using type of depression as the outcome variable, multivariate regression analysis revealed VCAM-1 to be a significant predictor of MDD (B = 0.003, 95% CI 0.001–0.007, *p* = 0.004) in the acute stage of the disorder (Table 3). VCAM-1 was also found to be a significant predictor in remission (B = 0.002, 95% CI: 0.0001–0.005, *p* = 0.012). ICAM-1 was not found to be a significant predictor in either the acute stage or remission.

**Figure 1:**
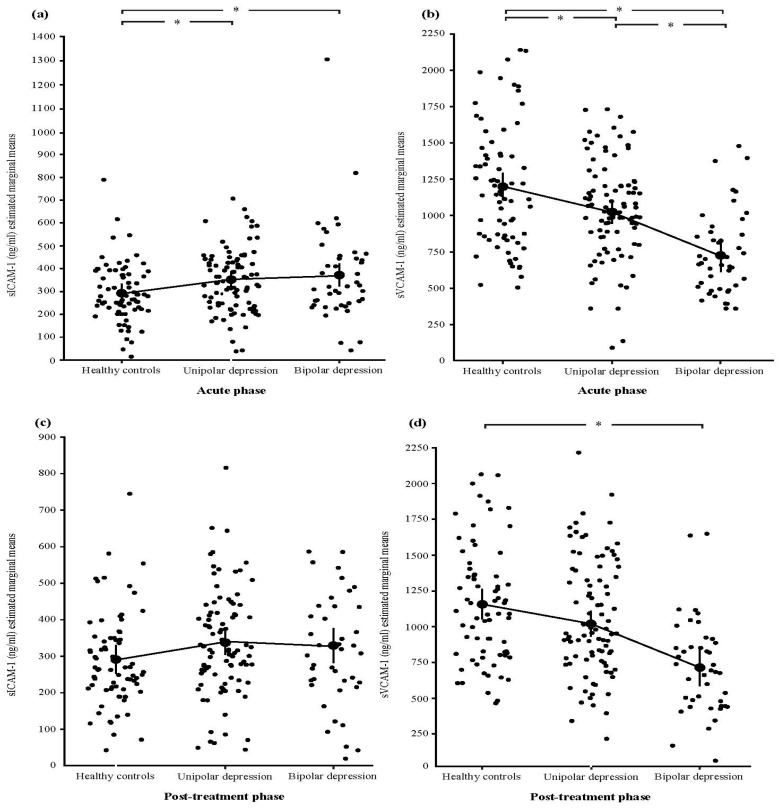
Differences in serum levels of soluble adhesion molecules sICAM-1 and sVCAM-1 among patients with unipolar and bipolar depression and healthy controls, before and after treatment application. (**a**) sICAM-1 values before treatment application (in the acute phases of the disorders) (UD: 349.05 ± 132.33; BD 383.91 ± 212.74; healthy controls 305.52 ± 132.59); (**b**) sVCAM-1 values before treatment application (in the acute phases of the disorders) (UD: 1047.79 ± 326.14; BD 762.79 ± 269.35; healthy controls 1193.36 ± 428.74); (**c**) sICAM-1 values after treatment application (UD: 346.53 ± 186.64; BD 343.19 ± 161.44; healthy controls 305.52 ± 132.59); (**d**) sVCAM-1 values after treatment application (UD: 1084.95 ± 476.36; BD 760.57 ± 335.60; healthy controls 1193.36 ± 428.74). * *p* < 0.05 and CI does not contain zero.

The serum concentrations of sICAM-1 and sVCAM-1 (pg/mL) of study subjects are presented in Figure 1.

### 3.2. Differences in sICAM-1 and sVCAM-1 between Patients with MDD and BD and Healthy Controls

Both patients in acute episodes of MDD (sICAM: *p* = 0.047, CI: 0.957–130.204; sVCAM: *p* = 0.022, CI: 30.773–324.240) and BD (sICAM: *p* = 0.018, CI: 13.886–144.333; sVCAM: *p* = 0.001, CI: 318.405–603.943) showed an imbalance of adhesion molecules when compared to the healthy control group (sICAM: F = 3.375, *p* = 0.037; sVCAM: F = 20.742, *p* < 0.001). The results also show down-regulation of sVCAM-1 and up-regulation of sICAM-1 levels in both patient groups (Figure 1).

When the effect of staging on the observed differences was analyzed, the results revealed that sICAM-1 concentrations in patients in the early stages of the disorders were similar to those of healthy subjects (*p* > 0.05), whereas only patients with late-stage UD, patients currently in an acute episode (*p* = 0.002, CI: 48.688–178.656), and patients with BD (*p* = 0.032, CI: 6.101–151.429) (F = 4.020, *p* = 0.004) had higher sICAM-1 levels. In contrast, sVCAM-1 levels were lower in both early (UD: *p* = 0.029, CI: 27.085–378.822; BD: *p* = 0.001, CI: 360.639–608.704) and late (UD: *p* = 0.045, CI: 2.882–328.034; BD: *p =* 0.001, CI: 407.503–666.400) stages of the disorders when compared to healthy controls (F = 21.613, *p* < 0.001).

**Figure 2:**
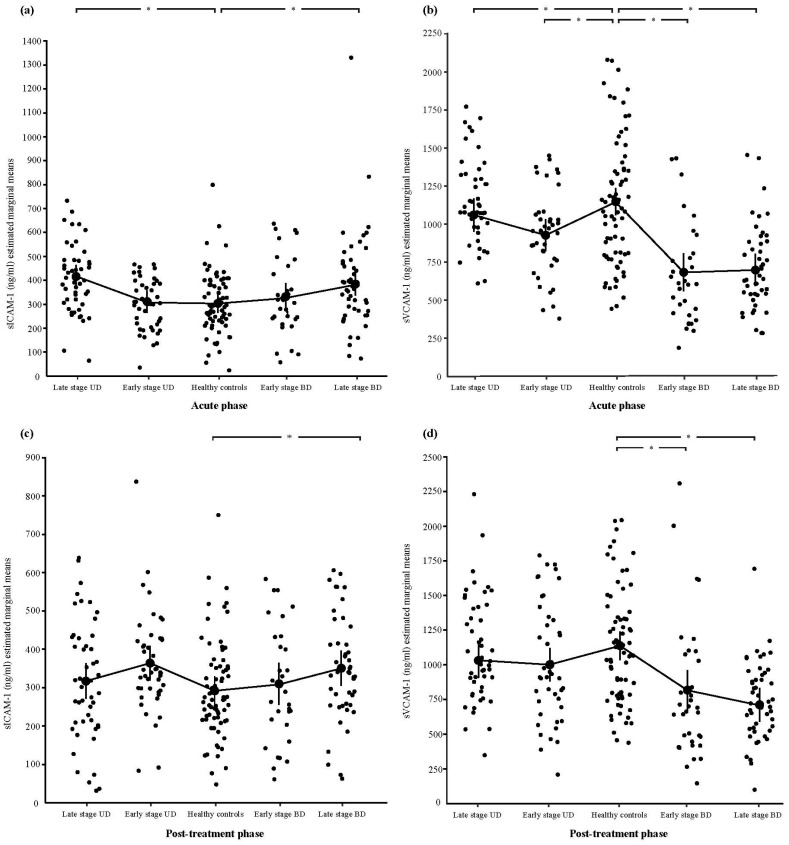
Serum levels of sICAM-1 and sVCAM-1 in early and late stages of UD and BD and healthy controls. (**a**) sICAM-1 values before treatment application (in the acute phases of the disorders); (**b**) sVCAM-1 values before treatment application (in the acute phases of the disorders); (**c**) sICAM-1 values after treatment application; (**d**) sVCAM-1 values after treatment application. * *p* < 0.05 and CI does not contain zero.

Furthermore, the main effects of diagnosis on the adhesion molecule markers were observed in sVCAM-1 (after covarying for multiple confounders—age, gender, smoking status, and BMI), showing decreased levels in patients with acute episodes of bipolar depression when compared to their unipolar counterparts (*p* = 0.002, CI: 155.103–423.843). The sVCAM-1 concentrations remained lower in both stages of BD when compared to their corresponding MDD levels (early stage: *p* = 0.001, CI: 97.207–465.384; late stage: *p* = 0.001, CI: 227.584–528.476). On the other hand, the levels of sICAM-1 were comparable between the patients with MDD and BD after adjusting for age, gender, smoking status, and BMI (*p* > 0.05), irrespective of their staging (*p* > 0.05) (Table 3, Figure 2).

### 3.3. Association between sICAM-1 and sVCAM-1 and Treatment Application

The results show that post-treatment sICAM-1 concentrations of both patient groups in remission were comparable to sICAM-1 concentrations in healthy subjects (F = 1.061, *p* = 0.349) (Figure 1). Further analysis according to the stage of the disorder, however, revealed that sICAM-1 normalized in all patient groups except for bipolar disorder patients in the late stage of illness (F = 1.387, *p* = 0.033, CI: 7.140–124.341) (Table 4).

On the other hand, post-treatment sVCAM-1 levels normalized only in the MDD patient group (*p* > 0.05), while they remained significantly lower in the group of patients with BD (*p* = 0.001, CI: 276.483–624.071) when compared to healthy controls (F = 13.359, *p* < 0.001) (Figure 1). This remained the same when patients were divided according to the stage of the disorder since sVCAM-1 levels stayed lower in patients in both early (*p* = 0.003, CI: 137.383–611.604) and late stages (*p* = 0.001, CI: 410.162–704.348) of bipolar disorder (F = 11.993, *p* < 0.001) (Table 4).

The analysis of treatment type and levels of immune markers showed no statistically significant associations between treatment application and sICAM-1 and sVCAM-1 concentrations in the whole sample or in the subsamples of MDD and BD (*p* > 0.05).

## 4. Discussion

To our knowledge, this is the first study to examine serum sICAM-1 and sVCAM-1 as potential tools to support clinical differences between patients with BD and those with MDD. It indicates that serum sVCAM-1 levels in BD are lower than in MDD and that sVCAM-1 may be a valuable immune mediator to differentiate between two depressive states. It also suggests the overall upregulation of sICAM-1 in MDD and BD and is the first to suggest the implication of these immune mediators in the staging of mood disorders. In addition, the results reveal a different post-treatment normalization pattern of immune markers between unipolar and bipolar depression.

The functional importance of imbalanced adhesion molecule levels in mood disorders is still unclear. The majority of previous studies reported either unchanged or elevated expression of VCAM-1 and ICAM-1, or their soluble forms, in major depression and bipolar disorder [7,17,22,23,41,42]. This is only partially in line with our results concerning an elevated soluble form of ICAM-1 in both disorders. The novelty of the present study is based on the fact that we have further explored the direct differences in multiple adhesion molecules between MDD and BD. Furthermore, previous studies predominantly refer to specific or limited populations of patients with mood disorders, including those in senium or those who died by a suicide attempt. In addition, we report lower sVCAM-1 levels in MDD and even more so in BD. Although the results concerning elevated sVCAM-1 levels are suggestive [13], the explored sVCAM-1 levels in healthy elderly controls in this study were two times lower than the ones we reported. Moreover, the sVCAM-1 levels in healthy controls reported in our sample are comparable to the majority of previous studies regardless of their design [8,43,44,45]. The observed differences could be due to a different biological medium or the fact that the study of Dimopoulos et al. [17] explored patients in late-life depression with various somatic disorders and did not calculate the effects of additional factors such as age and comorbidity. Also, as both sICAM-1 and sVCAM-1 play a role in BBB permeability, it is noteworthy that their soluble forms are not necessarily linearly related to their expression in the CNS [38]. However, the studies have also demonstrated that the peripheral concentration of immune mediators, although lower, could still represent the levels of these molecules in the CNS [46].

The present study showed down-regulated sVCAM-1 levels in both types of depression, which were even more distinct in the bipolar type, whereas immune differences in BD were more pronounced in patients with a previous family history of the same disorder. These findings indicate that the immune changes could mirror the high genetic load for bipolar disorder [44,47]. In our study, however, the BD group included a higher proportion of patients of female gender. Potentially, this may be attributed to possible depressive predominant polarity in this group. Not only is this polarity more frequent in female patients [47,48], but it could also be related to distinct immunological patterns [43], which should be considered when interpreting the results. Other studies also discuss more prominent low-grade inflammation in BD when compared to MDD [31,49]. This could be the reflection of a complex neuroimmune interaction and can be a marker of advanced apoptosis in mood disorders [50], particularly BD.

Elevated expression of adhesion molecules has been related to lymphocyte infiltration, which is known to increase neuronal excitability and promote neuroinflammation progression [51,52,53]. Since their soluble forms have also been marked as important indicators of severity and mental disorder progression, while being easy diagnostic markers in vivo, their similar roles in mood disorders are also to be expected [38,40]. Our findings related to the association of sICAM-1 with the late stages of both MDD and BD also support this notion. Likewise, mounting evidence from previous reports on overlapping immune dysfunction between MDD and BD point to a pronounced immune dysregulation in more advanced stages of mood disorders [46,54,55,56,57]. However, to our knowledge, this is the first study to explore sVCAM-1 as a specific stage biomarker in the progression of any mood disorder [37], with only one recent study exploring sICAM-1 in patients in a euthymic state of bipolar disorder [57]. Interestingly, high sICAM-1 levels have been previously predominantly reported in subsets of patients with late-life unipolar depression or in older patients with BD without exploring the longitudinal characteristics of the disorder [13,17,58]. Some studies found more pronounced inflammation in older patients and an association between the duration of illness and several proinflammatory mediators [16,59]. This could point to the delayed effects of adhesion molecule-mediated inflammation on disease progression [60].

The novelty of our results concerning the importance of sICAM-1 in staging the disorder is further confirmed by a recent study assessing the different stages of illness in patients with schizophrenia and euthymic bipolar disorder [34,57]. Results from other studies indicate that inflammatory processes in the brain are further challenged by prolonged stress, leading to increased allostatic load in patients with mood disorders [61,62]. The disbalance of sICAM-1, observed in both late-stage patient groups in the study, coupled with particular upregulation in those with a longer course of ongoing exposure to illness symptoms further supports the hypothesis of allostatic load as crucial in longitudinal development of psychiatric and, particularly, mood disorders.

The present results linking sVCAM-1 with the number of mood episodes in the overall sample further contribute to this hypothesis. Although the relationship was not confirmed in subsamples of patients with MDD or BD, probably due to a smaller sample size, it could be a reason for lower sVCAM-1 in BD. Clinical studies consider bipolar disorder as a more severe disorder associated with an earlier onset, longer bouts of time spent ill, and higher recurrence than unipolar disorder [45,63,64]. To that end, the observed down-regulation of sVCAM-1 in BD could be a reliable epiphenomenon of severity and a more pronounced staging of the disorder caused by multiple neuroimmune disturbances associated with higher recurrence and increased illness burden [7]. This is of particular clinical importance, bearing in mind our study included patients who required hospital treatment and thus, potentially, those with generally more severe forms of the disorder exposed to more frequent subthreshold symptoms and with decreased interepisode functioning.

Our study found not only that sVCAM-1 can differentiate between the disorders but also that post-treatment biomarker levels normalize only in patients with MDD. Together with the previously discussed findings, these data suggest that inflammation could be associated with constitutional molecular characteristics further challenged by factors that may contribute to a higher allostatic load. This could lead to a maintained proinflammatory state in euthymia and irreversible structural changes, preventing the restoration of homeostasis in patients with bipolar disorder. However, the study of Schaefer et al. [23] did not find normalization of upregulated sICAM-1 in MDD. We have included a larger sample size, allowing us to further assess patients in different stages of the disorder. Consequently, our study was the first to indicate sICAM-1 normalization in both patient groups regardless of the stage of the disorder. Both Rowland et al. and Berk et al. [46,65] found that patients with mood disorders in acute exacerbation of the illness have higher concentrations of immune mediators compared to healthy controls. Interestingly, the same studies reported that patients in the remission period seemed to either reach normal levels of immune mediators or maintain a proinflammatory state. This is also in line with our results concerning post-treatment levels of sICAM-1 and, partially, sVCAM-1.

sICAM-1 appears to be upregulated in many psychiatric and neurodegenerative disorders or after brain injury. Expressed by endothelial cells, it can be considered an indicator of damaged vascular integrity and increased cell trafficking through the BBB. In inflammatory conditions, it is upregulated and fosters leukocyte migration into the CNS in an attempt to limit the inflammation [66]. The same is noted both in our results and previous studies. However, the post-treatment downregulation of sICAM-1 observed in our study could be a compensatory phenomenon, indicating decreased leucocyte trafficking through the BBB and a struggle to counteract the pro-inflammatory pathway [21]. Similarly, downregulation of sVCAM-1 could provide further support, as reduced sVCAM-1 levels were also found to be indicators of disorder progression and poor outcome in cancer patients [67]. Similarly, in our patients, reduced sVCAM-1 levels could present an initial mechanism to mitigate the inflammation, reduce cell trafficking, and restore BBB integrity in a response to previously enhanced endothelial expression of VCAM-1 [68].

The interpretation of our findings would benefit from taking into account some of the limitations and strengths of the study. The treatment between the groups differed as it was applied according to contemporary guidelines. The effects of treatment on serum immune markers, therefore, should not be forgotten because the observed disbalance may be the consequence of different treatments between the study groups or even dose-dependent, longitudinal exposure to medication. Also, the unbalanced proportion of sexes in study groups, particularly in the bipolar depression group, warrants cautious interpretation of the obtained results. Although our study did not show sex differences in adhesion molecules regardless of the patient groups, previous studies have extensively discussed the association between sex and inflammation [69,70]. It should be pointed out, however, that our study controlled for potential confounding effects of gender in all analyses. In addition, due to the previously reported high conversion rate from unipolar to bipolar disorder [61], we are prevented from extending our findings to the whole population. However, the study applied a strict selection method to assess the polarity of the episode and appropriate clinical staging classification, thus meeting the restrictions of limited previous studies [7,23,26,31]. We also included a higher number of patients and allowed comparison with healthy control groups. The study also explored both acute and longitudinal characteristics of the disorder and accounted for the effects of important potential confounding factors.

## 5. Conclusions

Our study presents what may be the first investigation into the diagnostic potential of different adhesion molecules for MDD and BD. The data indicate adhesion molecules, especially sVCAM-1, serve as promising indicators of the distinctive cellular adhesion activities present in MDD and BD. These molecules might serve as tools for distinguishing between BD and MDD patients, and they appear to be elements within the intricate inflammatory network that contributes to the shared pathogenetic foundation and the advancement of these conditions. The findings reinforce the notion that BD could be linked to a more profound inflammatory imbalance. Additionally, the current nosology of mood disorders tends to offer a static, categorical view of these illnesses, neglecting their time-dependent pathoplasticity and biological correlates. To further understand the extent to which our results represent inherent anomalies, the consequences of disease progression, or clinical outcomes, future research should incorporate biomarkers into broad, prospective cohorts.

## Figures and Tables

**Table 1:** Socio-demographic and clinical characteristics of the study groups.

Socio-Demographic Variables	BD (*n* = 44)	UD (*n* = 93)	Healthy Controls (*n* = 73)	Statistics	*p*-Values
Sex (female, %)	93.70	64.10	56.80	χ^2^ = 13.430	0.001 ^a^
Age (years)	46.62 ± 9.76	47.54 ± 8.59	45.82 ± 8.19	F = 20.550	0.291 ^b^
BMI (kg/m^2^)	24.51 ± 4.05	23.94 ± 2.36	24.98 ± 3.79	F = 11.383	0.163 ^b^
Smoker (yes, %)	65.10	56.50	48.60	χ^2^ = 3.075	0.215 ^a^
Marital state (with partner, %)	60.50	64.70	61.10	χ^2^ = 0.221	0.638 ^a^
Education (years)	12.28 ± 2.16	11.28 ± 2.18	14.38 ± 2.44	F = 122.335	<0.001 ^b^
Employed (%)	30.20	62.40	87.30	χ^2^ = 38.314	<0.001 ^a^
**Clinical Variables**					
Age of onset (years)	29.74 ± 9.57	39.39 ± 11.01	-	t = −4.870	<0.001 ^c^
Duration of illness (years)	18.76 ± 10.93	12.96 ± 8.68	-	t = 3.299	0.001 ^c^
Number of previous episodes	11.11 ± 7.80	5.10 ± 3.00	-	t = 6.378	<0.001 ^c^
Number of previous inpatient treatments	8.74 ± 7.96	3.98 ± 3.18	-	t = 4.906	<0.001 ^c^
Duration of current episode (months)	2.05 ± 1.02	2.92 ± 1.47	-	t = −3.475	0.001 ^a^
Duration of untreated disorder (months)	113.86 ± 94.49	47.97 ± 53.16	-	t = 5.122	<0.001 ^c^
Early-stage disorder (%)	43.18	48.39	-	χ^2^ = 1.247	0.176 ^a^
HDRS—Acute phase	27.33 ± 10.30	23.85 ± 5.62	-	t = 5.113	<0.001 ^c^
HDRS—Remission phase	4.98 ± 2.25	3.56 ± 3.07	-	t = 3.285	0.001 ^c^
YMRS—Acute phase	5.02 ± 1.42	3.22 ± 2.04	-	t = 4.734	<0.001 ^c^
YMRS—Remission phase	1.42 ± 1.91	1.56 ± 1.22	-	t = 2.190	0.231 ^c^
CGI-S	4.86 ± 0.82	4.62 ± 0.44	-	t = 4.906	0.381 ^c^
Applied treatment (%)					
Mood stabilizer	72.72	11.83	-	χ^2^ = 51.089	<0.001 ^d^
Antidepressant	45.45	100.00	-	χ^2^ = 57.784	<0.001 ^d^
Antypsychotic	56.81	17.20	-	χ^2^ = 20.502	<0.001 ^d^
Psychiatric heredity (yes, %)	63.63	43.01	-	χ^2^ = 4.291	0.038 ^d^
Heredity for the same disorder	4.70	31.18	-	-	<0.001 ^e^
Psychotic episode (yes, %)	36.36	17.20	-	χ^2^ = 5.101	0.024 ^d^
Time to remission (days)	36.09 ± 14.16	31.07 ± 10.47	-	t = 2.268	0.025 ^c^

All values are means ± standard deviations, except where otherwise specified. (a) Chi-square test; (b) one-way analyses of variance (ANOVA); (c) independent sample *t*-test/Mann–Whitney U test, depending on data normality; (d) Chi-square test with Yates correction for continuity; (e) Fisher’s exact test. Abbreviations: BD = bipolar depression; BMI = body mass index; CGI-S = Clinical Global Impression Scale—Severity; UD = unipolar depression; HDRS = Hamilton Depression Rating Scale; YMRS = Young Mania Rating Scale.

**Table 2:** Partial correlation coefficients of clinical characteristics and the type of disorder.

	BD and UD				BD				UD			
Course of Illness Characteristics	sVCAM-1		sICAM-1		sVCAM-1		sICAM-1		sVCAM-1		sICAM-1	
	A	R	A	R	A	R	A	R	A	R	A	R
Age of onset (years)	0.170	0.136	−0.026	−0.069	0.249	0.290	0.232	0.112	−0.002	−0.364	−0.127	−0.151
Duration of illness (years)	−0.190	−0.190	0.146	0.028	−0.212	−0.262	−0.152	−0.086	−0.016	0.316	0.394 ***	0.127
Number of episodes, total	−0.272 **	−0.190	−0.120	0.017	−0.177	0.123	−0.182	−0.072	0.048	−0.104	0.030	−0.041
Number of previous inpatient treatments	−0.230 *	−0.123	−0.079	0.016	−0.149	0.169	−0.176	−0.125	0.027	−0.111	0.033	−0.11
Duration of current episode (months)	0.087	−0.054	0.167	−0.083	−0.286	−0.237	0.042	−0.332	0.029	−0.026	0.317 **	0.152
Duration of untreated disorder (months)	−0.186	−0.256	−0.167	−0.176	−0.125	−0.230	−0.227	−0.274	−0.013	−0.034	0.141	−0.193
HDRS—Acute phase	−0.180	−0.060	−0.004	−0.002	0.072	0.148	0.357 *	0.190	−0.203	−0.282	−0.031	−0.209
HDRS—Remission phase	−0.033	0.192	0.045	−0.079	−0.037	0.175	0.039	−0.030	−0.029	0.098	0.043	−0.032
YMRS—Acute phase					0.007	−0.056	−0.299	−0.313				
YMRS—Remission phase					−0.059	−0.125	−0.257	−0.245				
CGI-S	−0.117	−0.156	0.034	−0.195	−0.207	−0.188	0.004	−0.115	−0.109	−0.113	0.044	−0.201
Time to remission (days)	−0.266 *	−0.114	−0.127	−0.015	−0.137	0.148	−0.097	−0.155	−0.227	−0.144	0.041	0.139

Abbreviations: BD = bipolar depression; UD = unipolar depression; sVCAM-1 = soluble vascular cell adhesion molecule-1; sICAM-1 = soluble intercellular adhesion molecule-1; CGI-S = Clinical Global Impression Scale—Severity; UD = unipolar depression; HDRS = Hamilton Depression Rating Scale; YMRS = Young Mania Rating Scale. * *p* < 0.05, ** *p* < 0.01, *** *p* < 0.001.

**Table 3:** Multivariate regression analysis of predictors for the type of disorder.

Variables	Multivariate Regression		
**Acute Phase**	**B**	**95% CI**	***p***
Sex (female)	−1.772	−27.718 to −0.793	0.002
Age (years)	0.066	0.019–0.140	0.011
BMI	0.349	−0.871 to −0.128	0.10
Smoking	−0.393	−1.515 to 0.687	0.416
ICAM-1	0.001	−0.002–0.005	0.558
Sex (female)	−1.62	−20.41 to −0.37	0.110
Age (years)	0.036	−0.029–0.118	0.244
BMI	−0.274	−0.693 to −0.090	0.17
Smoking	0.317	−0.873–1.790	0.621
VCAM-1	0.003	0.001–0.007	0.004
**Remission**	**B**	**95% CI**	***p***
Sex (female)	−1.227	−21.182–0.096	0.046
Age (years)	0.075	0.016–0.177	0.012
BMI	−0.240	−0.655 to −0.027	0.047
Smoking	−0.200	−1.609–1.411	0.757
ICAM-1	0.002	−0.002–0.006	0.282
Sex (female)	−1.056	−22.387–0.909	0.189
Age (years)	0.035	−0.043–0.133	0.332
BMI	−0.188	−0.585 to −0.03	0.084
Smoking	0.234	−1.501–2.121	0.736
VCAM-1	0.002	0.0001–0.005	0.012

Abbreviations: sVCAM-1 = soluble vascular cell adhesion molecule-1; sICAM-1 = soluble intercellular adhesion molecule-1; BMI = body mass index. CI = confidence interval.

**Table 4:** Serum levels of sICAM-1 and sVCAM-1 in early and late stages of UD and BD and healthy controls.

Immune Mediator	Unipolar Depression (UD)		Bipolar Depression (BD)		Healthy Controls	UD vs. HC (*p*) ^a^		BD vs. HC (*p*) ^a^	
	Early Stage	Late Stage	Early Stage	Late Stage		Early Stage	Late Stage	Early Stage	Late Stage
Pre-treatment sICAM-1	301.80 ± 113.34	402.02 ± 133.54	332.83 ± 159.65	387.87 ± 196.24	305.52 ± 132.59	0.803	0.002 *	0.534	0.032 *
Post-treatment sICAM-1	362.88 ± 167.63	324.71 ± 213.48	320.65 ± 168.34	376.93 ± 136.39	305.52 ± 132.59	0.114	0.664	0.779	0.033
Pre-treatment sVCAM-1	992.82 ± 376.42	1095.88 ± 271.88	675.06 ± 234.36	687.82 ± 111.02	1193.36 ± 428.74	0.029 *	0.045 *	0.001 *	0.001 *
Post-treatment sVCAM-1	1065.82 ± 460.33	1121.09 ± 532.05	757.64 ± 515.10	696.01 ± 294.36	1193.36 ± 428.74	0.358	0.527	0.003 *	0.001 *

All values are means ± standard deviations, except where otherwise specified. (**a**) Analyses of co-variance (ANCOVA) with covariates: age, gender, BMI, smoking status. * *p* < 0.05 and CI does not contain zero. Abbreviations: UD = unipolar depression; BD = bipolar depression.

## Data Availability

The datasets used and/or analysed during the current study are available from the corresponding author on reasonable request.

## References

[1] Brunoni A.R., Supasitthumrong T., Teixeira A.L., Vieira E.L., Gattaz W.F., Benseñor I.M., Lotufo P.A., Lafer B., Berk M., Carvalho A.F. (2020). Differences in the Immune-Inflammatory Profiles of Unipolar and Bipolar Depression. J. Affect. Disord..

[2] Hori H., Teraishi T., Sasayama D., Hattori K., Hashikura M., Higuchi T., Kunugi H. (2013). Relationship of Temperament and Character with Cortisol Reactivity to the Combined Dexamethasone/CRH Test in Depressed Outpatients. J. Affect. Disord..

[3] Kapczinski F., Magalhães P.V.S., Balanzá-Martinez V., Dias V.V., Frangou S., Gama C.S., Gonzalez-Pinto A., Grande I., Ha K., Kauer-Sant’Anna M. (2014). Staging Systems in Bipolar Disorder: An International Society for Bipolar Disorders Task Force Report. Acta Psychiatr. Scand..

[4] Millar K., Lloyd S.M., McLean J.S., Batty G.D., Burns H., Cavanagh J., Deans K.A., Ford I., McConnachie A., McGinty A. (2013). Personality, Socio-Economic Status and Inflammation: Cross-Sectional, Population-Based Study. PLoS ONE.

[5] Rowland T., Perry B.I., Upthegrove R., Barnes N., Chatterjee J., Gallacher D., Marwaha S. (2018). Neurotrophins, Cytokines, Oxidative Stress Mediators and Mood State in Bipolar Disorder: Systematic Review and Meta-Analyses. Br. J. Psychiatry.

[6] Hashimoto K. (2015). Brain-Derived Neurotrophic Factor (BDNF) and Its Precursor ProBDNF as Diagnostic Biomarkers for Major Depressive Disorder and Bipolar Disorder. Eur. Arch. Psychiatry Clin. Neurosci..

[7] Thomas A.J., Davis S., Ferrier I.N., Kalaria R.N., O’Brien J.T. (2004). Elevation of Cell Adhesion Molecule Immunoreactivity in the Anterior Cingulate Cortex in Bipolar Disorder. Biol. Psychiatry.

[8] Turan Ç., Kesebir S., Süner Ö. (2014). Are ICAM, VCAM and E-Selectin Levels Different in First Manic Episode and Subsequent Remission?. J. Affect. Disord..

[9] Müller N., Riedel M., Hadjamu M., Schwarz M.J., Ackenheil M., Gruber R. (1999). Increase in expression of adhesion molecule receptors on T helper cells during antipsychotic treatment and relationship to bloodbrain barrier permeability in schizophrenia. Am. J. Psychiatry.

[10] Lassmann H., Rössler K., Zimprich F., Vass K. (1991). Expression of adhesion molecules and histocompatibility antigens at the blood-brain barrier. Brain Pathol..

[11] Dietrich J.B. (2002). The adhesion molecule ICAM-1 and its regulation in relation with the blood-brain barrier. J. Neuroimmunol..

[12] Wu F., Liu L., Zhou H. (2017). Endothelial cell activation in central nervous system inflammation. J. Leukoc. Biol..

[13] Cai H.Q., Catts V.S., Webster M.J., Galletly C., Liu D., O’Donnell M., Weickert T.W., Weickert C.S. (2020). Increased macrophages and changed brain endothelial cell gene expression in the frontal cortex of people with schizophrenia displaying inflammation. Mol. Psychiatry.

[14] Ormel P.R., Böttcher C., Gigase F.A.J., Missall R.D., van Zuiden W., Fernández Zapata M.C., Ilhan D., de Goeij M., Udine E., Sommer I.E.C. (2020). A characterization of the molecular phenotype and inflammatory response of schizophrenia patient derived microglia-like cells. Brain Behav. Immun..

[15] Berry M., Logan A. (2019). CNS Injuries Cellular Responses and Pharmacological Strategies.

[16] Spampinato S.F., Merlo S., Fagone E., Fruciano M., Barbagallo C., Kanda T., Sano Y., Purrello M., Vancheri C., Ragusa M. (2019). Astrocytes Modify Migration of Pbmcs Induced by β-Amyloid in a Blood-Brain Barrier in Vitro Model. Front. Cell. Neurosci..

[17] Dimopoulos N., Piperi C., Salonicioti A., Mitsonis C., Liappas I., Lea R.W., Kalofoutis A. (2006). Elevation of Plasma Concentration of Adhesion Molecules in Late-Life Depression. Int. J. Geriatr. Psychiatry.

[18] O’Sullivan J.B., Ryan K.M., Harkin A., Connor T.J. (2010). Noradrenaline Reuptake Inhibitors Inhibit Expression of Chemokines IP-10 and RANTES and Cell Adhesion Molecules VCAM-1 and ICAM-1 in the CNS Following a Systemic Inflammatory Challenge. J. Neuroimmunol..

[19] Saengjaroentham C., Supornsilpchai W., Ji-Au W., Srikiatkhachorn A., Maneesri-Le Grand S. (2014). Serotonin Depletion Can Enhance the Cerebrovascular Responses Induced by Cortical Spreading Depression via the Nitric Oxide Pathway. Int. J. Neurosci..

[20] Meixensberger S., Kuzior H., Fiebich B.L., Süß P., Runge K., Berger B., Nickel K., Denzel D., Schiele M.A., Michel M. (2021). Upregulation of sICAM-1 and sVCAM-1 Levels in the Cerebrospinal Fluid of Patients with Schizophrenia Spectrum Disorders. Diagnostics.

[21] Müller N. (2019). The Role of Intercellular Adhesion Molecule-1 in the Pathogenesis of Psychiatric Disorders. Front. Pharmacol..

[22] Miguel-Hidalgo J.J., Overholser J.C., Jurjus G.J., Meltzer H.Y., Dieter L., Konick L., Stockmeier C.A., Rajkowska G. (2011). Vascular and Extravascular Immunoreactivity for Intercellular Adhesion Molecule 1 in the Orbitofrontal Cortex of Subjects with Major Depression: Age-Dependent Changes. J. Affect. Disord..

[23] Schaefer M., Sarkar S., Schwarz M., Friebe A. (2016). Soluble Intracellular Adhesion Molecule-1 in Patients with Unipolar or Bipolar Affective Disorders: Results from a Pilot Trial. Neuropsychobiology.

[24] Serebruany V.L., Suckow R.F., Cooper T.B., O’Connor C.M., Malinin A.I., Krishnan K.R., van Zyl L.T., Lekht V., Glassman A.H. (2005). Sertraline Antidepressant Heart Attack Randomized Trial. Relationship between release of platelet/endothelial biomarkers and plasma levels of sertraline and N-desmethylsertraline in acute coronary syndrome patients receiving SSRI treatment for depression. Am. J. Psychiatry.

[25] van Zyl L.T., Lespérance F., Frasure-Smith N., Malinin A.I., Atar D., Laliberté M.A., Serebruany V.L. (2009). Platelet and endothelial activity in comorbid major depression and coronary artery disease patients treated with citalopram: The Canadian Cardiac Randomized Evaluation of Antidepressant and Psychotherapy Efficacy Trial (CREATE) biomarker sub-study. J. Thromb. Thrombolysis.

[26] Yu B., Becnel J., Zerfaoui M., Rohatgi R., Boulares A.H., Nichols C.D. (2008). Serotonin 5-hydroxytryptamine(2A) receptor activation suppresses tumor necrosis factor-alpha-induced inflammation with extraordinary potency. J. Pharmacol. Exp. Ther..

[27] Schwarz M.J., Riedel M., Ackenheil M., Müller N. (2000). Decreased Levels of Soluble Intercellular Adhesion Molecule-1 (SICAM-1) in Unmedicated and Medicated Schizophrenic Patients. Biol. Psychiatry.

[28] American Psychiatric Association (2013). Diagnostic and Statistical Manual of Mental Disorders.

[29] Hirschfeld R.M.A., Williams J.B.W., Spitzer R.L., Calabrese J.R., Flynn L., Keck J., Lewis L., McElroy S.L., Post R.M., Rapport D.J. (2000). Development and Validation of a Screening Instrument for Bipolar Spectrum Disorder: The Mood Disorder Questionnaire. Am. J. Psychiatry.

[30] Hamilton M. (1967). Development of a Rating Scale for Primary Depressive Illness. Br. J. Soc. Clin. Psychol..

[31] Young R.C., Biggs J.T., Ziegler V.E., Meyer D.A. (1978). A Rating Scale for Mania: Reliability, Validity and Sensitivity. Br. J. Psychiatry.

[32] Guy W. (1976). ECDEU Assessment Manual for Psychopharmacology.

[33] Spearing M.K., Post R.M., Leverich G.S., Brandt D., Nolen W. (1997). Modification of the Clinical Global Impressions (CGI) Scale for Use in Bipolar Illness (BP): The CGI-BP. Psychiatry Res..

[34] Kapczinski F., Dias V.V., Kauer-Sant’Anna M., Brietzke E., Vázquez G.H., Vieta E., Berk M. (2009). The Potential Use of Biomarkers as an Adjunctive Tool for Staging Bipolar Disorder. Prog. Neuropsychopharmacol. Biol. Psychiatry.

[35] Dawood T., Barton D.A., Lambert E.A., Eikelis N., Lambert G.W. (2016). Examining Endothelial Function and Platelet Reactivity in Patients with Depression before and after SSRI Therapy. Front. Psychiatry.

[36] Durgam S., Starace A., Li D., Migliore R., Ruth A., Németh G., Laszlovszky I. (2015). The Efficacy and Tolerability of Cariprazine in Acute Mania Associated with Bipolar I Disorder: A Phase II Trial. Bipolar Disord..

[37] Tohen M., Frank E., Bowden C.L., Colom F., Ghaemi S.N., Yatham L.N., Malhi G.S., Calabrese J.R., Nolen W.A., Vieta E. (2009). The International Society for Bipolar Disorders (ISBD) Task Force Report on the Nomenclature of Course and Outcome in Bipolar Disorders. Bipolar Disord..

[38] Witkowska A.M., Borawska M.H. (2004). Soluble Intercellular Adhesion Molecule-1 (SICAM-1): An Overview. Eur. Cytokine Netw..

[39] Lynch D.F., Hassen W., Clements M.A., Schellhammer P.F., Wright G.L. (1997). Serum levels of endothelial and neural cell adhesion molecules in prostate cancer. Prostate.

[40] Stefanović M.P., Petronijević N., Dunjić-Kostić B., Velimirović M., Nikolić T., Jurišić V., Lačković M., Damjanović A., Totić-Poznanović S., Jovanović A.A. (2016). Role of SICAM-1 and SVCAM-1 as Biomarkers in Early and Late Stages of Schizophrenia. J. Psychiatr. Res..

[41] Thomas A.J., Ferrier I.N., Kalaria R.N., Davis S., O’Brien J.T. (2002). Cell Adhesion Molecule Expression in the Dorsolateral Prefrontal Cortex and Anterior Cingulate Cortex in Major Depression in the Elderly. Br. J. Psychiatry.

[42] Thomas A.J., Perry R., Kalaria R.N., Oakley A., McMeekin W., O’Brien J.T. (2003). Neuropathological Evidence for Ischemia in the White Matter of the Dorsolateral Prefrontal Cortex in Late-Life Depression. Int. J. Geriatr. Psychiatry.

[43] Kobawala T.P., Trivedi T.I., Gajjar K.K., Patel D.H., Patel G.H., Ghosh N.R. (2016). Significance of TNF-α and the Adhesion Molecules: L-Selectin and VCAM-1 in Papillary Thyroid Carcinoma. J. Thyroid Res..

[44] Pantović-Stefanović M., Petronijević N., Dunjić-Kostić B., Velimirović M., Nikolić T., Jurišić V., Lačković M., Damjanović A., Totić-Poznanović S., Jovanović A.A. (2016). SVCAM-1, SICAM-1, TNF-α and IL-6 Levels in Bipolar Disorder Type I: Acute, Longitudinal and Therapeutic Implications. Biol. Psychiatry.

[45] Tchalla A.E., Wellenius G.A., Sorond F.A., Travison T.G., Dantoine T., Lipsitz L.A. (2015). Elevated Circulating Vascular Cell Adhesion Molecule-1 (SVCAM-1) Is Associated with Concurrent Depressive Symptoms and Cerebral White Matter Hyperintensities in Older Adults Biology and Technology. BMC Geriatr..

[46] Rosenblat J.D., McIntyre R.S. (2017). Bipolar Disorder and Immune Dysfunction: Epidemiological Findings, Proposed Pathophysiology and Clinical Implications. Brain Sci..

[47] Baldessarini R.J., Undurraga J., Vázquez G.H., Tondo L., Salvatore P., Ha K., Khalsa H.M.K., Lepri B., Ha T.H., Chang J.S. (2012). Predominant recurrence polarity among 928 adult international bipolar I disorder patients. Acta Psychiatr. Scand..

[48] Nivoli A.M.A., Pacchiarotti I., Rosa A.R., Popovic D., Murru A., Valenti M., Bonnin C.M., Grande I., Sanchez-Moreno J., Vieta E. (2011). Gender differences in a cohort study of 604 bipolar patients: The role of predominant polarity. J. Affect. Disord..

[49] Bai Y.M., Su T.P., Li C.T., Tsai S.J., Chen M.H., Tu P.C., Chiou W.F. (2015). Comparison of Pro-Inflammatory Cytokines among Patients with Bipolar Disorder and Unipolar Depression and Normal Controls. Bipolar Disord..

[50] Rose D.M., Cardarelli P.M., Cobb R.R., Ginsberg M.H. (2000). Soluble VCAM-1 Binding to A4 Integrins Is Cell-Type Specific and Activation Dependent and Is Disrupted during Apoptosis in T Cells. Blood.

[51] Chatterjee M., Schild D., Teunissen C. (2019). Contactins in the Central Nervous System: Role in Health and Disease. Neural Regen. Res..

[52] Larochelle C., Alvarez J.I., Prat A., Williams R., Flügel A., Just W. (2011). How Do Immune Cells Overcome the Blood–Brain Barrier in Multiple Sclerosis?. FEBS Lett..

[53] Rana A., Musto A.E. (2018). The Role of Inflammation in the Development of Epilepsy. J. Neuroinflamm..

[54] Castaño-Ramírez O.M., Sepúlveda-Arias J.C., Duica K., Díaz Zuluaga A.M., Vargas C., López-Jaramillo C. (2018). Inflammatory Markers in the Staging of Bipolar Disorder: A Systematic Review of the Literature. Rev. Colomb. Psiquiatr. (Engl. Ed.).

[55] Maes M., Mihaylova I., Kubera M., Ringel K. (2012). Activation of Cell-Mediated Immunity in Depression: Association with Inflammation, Melancholia, Clinical Staging and the Fatigue and Somatic Symptom Cluster of Depression. Prog. Neuropsychopharmacol. Biol. Psychiatry.

[56] Wysokiński A., Szczepocka E. (2016). Platelet Parameters (PLT, MPV, P-LCR) in Patients with Schizophrenia, Unipolar Depression and Bipolar Disorder. Psychiatry Res..

[57] Maes M.H., Stoyanov D. (2022). False dogmas in mood disorders research: Towards a nomothetic network approach. World J. Psychiatry.

[58] Maes M., Moraes J.B., Bonifacio K.L., Barbosa D.S., Vargas H.O., Michelin A.P., Nunes S.O.V. (2021). Towards a new model and classification of mood disorders based on risk resilience, neuro-affective toxicity, staging, and phenome features using the nomothetic network psychiatry approach. Metab. Brain Dis..

[59] Van Sloten T.T., Schram M.T., Adriaanse M.C., Dekker J.M., Nijpels G., Teerlink T., Scheffer P.G., Pouwer F., Schalkwijk C.G., Stehouwer C.D.A. (2014). Endothelial Dysfunction Is Associated with a Greater Depressive Symptom Score in a General Elderly Population: The Hoorn Study. Psychol. Med..

[60] Osimo E.F., Pillinger T., Rodriguez I.M., Khandaker G.M., Pariante C.M., Howes O.D. (2020). Inflammatory Markers in Depression: A Meta-Analysis of Mean Differences and Variability in 5166 Patients and 5083 Controls. Brain Behav. Immun..

[61] Giovannoni G., Miller D.H., Losseff N.A., Sailer M., Lewellyn-Smith N., Thompson A.J., Thompson E.J. (2001). Serum Inflammatory Markers and Clinical/MRI Markers of Disease Progression in Multiple Sclerosis. J. Neurol..

[62] Jeon S.W., Kim Y.K. (2018). The Role of Neuroinflammation and Neurovascular Dysfunction in Major Depressive Disorder. J. Inflamm. Res..

[63] Wolkowitz O.M., Mellon S.H., Epel E.S., Lin J., Dhabhar F.S., Su Y., Reus V.I., Rosser R., Burke H.M., Kupferman E. (2011). Leukocyte Telomere Length in Major Depression: Correlations with Chronicity, Inflammation and Oxidative Stress-Preliminary Findings. PLoS ONE.

[64] Goodwin F.K., Jamison K.R. (2007). Manic-Depressive Illness: Bipolar Disorders and Recurrent Depression.

[65] Motovsky B., Pecenak J. (2013). Psychopathological characteristics of bipolar and unipolar depression-potential indicators of bipolarity. Psychiatr. Danub..

[66] Berk M., Williams L.J., Jacka F.N., O’Neil A., Pasco J.A., Moylan S., Allen N.B., Stuart A.L., Hayley A.C., Byrne M.L. (2013). So Depression Is an Inflammatory Disease, but Where Does the Inflammation Come From?. BMC Med..

[67] Okugawa Y., Miki C., Toiyama Y., Koike Y., Yokoe T., Saigusa S., Tanaka K., Inoue Y., Kusunoki M. (2010). Soluble VCAM-1 and its relation to disease progression in colorectal carcinoma. Exp. Ther. Med..

[68] Brishti M.A., Raghavan S., Lamar K., Singh U.P., Collier D.M., Leo M.D. (2023). Diabetic Endothelial Cell Glycogen Synthase Kinase 3β Activation Induces VCAM1 Ectodomain Shedding. Int. J. Mol. Sci..

[69] Niles A.N., Smirnova M., Lin J., O’Donovan A. (2018). Gender differences in longitudinal relationships between depression and anxiety symptoms and inflammation in the health and retirement study. Psychoneuroendocrinology.

[70] Trabace L., Roviezzo F., Rossi A. (2022). Editorial: Sex Differences in Inflammatory Diseases. Front. Pharmacol..

